# Angiogenesis in pancreatic ductal adenocarcinoma: A controversial issue

**DOI:** 10.18632/oncotarget.10765

**Published:** 2016-07-21

**Authors:** Vito Longo, Oronzo Brunetti, Antonio Gnoni, Stefano Cascinu, Giampietro Gasparini, Vito Lorusso, Domenico Ribatti, Nicola Silvestris

**Affiliations:** ^1^ Department of Medical Oncology, Hospital of Taranto, Taranto, Italy; ^2^ Medical Oncology Unit, Cancer Institute “Giovanni Paolo II”, Bari, Italy; ^3^ Department of Medical Oncology, Hospital “Vito Fazi” of Lecce, Lecce, Italy; ^4^ Medical Oncology Unit, University of Modena, Modena, Italy; ^5^ Scientific Direction, Cancer Institute “Giovanni Paolo II”, Bari, Italy; ^6^ Department of Basic Medical Sciences, Neurosciences and Sensory Organs, University of Bari Medical School, Bari, Italy; ^7^ National Cancer Institute “Giovanni Paolo II”, Bari, Italy

**Keywords:** angiogenesis, pancreatic ductal adenocarcinoma, hypoxia, desmoplastic reaction

## Abstract

Pancreatic ductal adenocarcinoma (PDAC) occurs in the majority of cases with early loco-regional spread and distant metastases at diagnosis, leading to dismal prognosis with a 5-year overall survival rate moderately over than 5%. This malignancy is largely resistant to chemotherapy and radiation, but the reasons of the refractoriness to the therapies is still unknown. Evidence is accumulating to indicate that the PDAC microenvironment and vascularity strongly contribute to the clinical features of this disease. In particular, PDAC is characterized by excessive dense extracellular matrix deposition associated to vasculature collapse and hypoxia with low drug delivery, explaining at least partly the low efficacy of antiangiogenic drugs in this cancer. Strategies aimed to modulate tumor stroma favoring vasculature perfusion and chemotherapeutics delivery are under investigation.

## INTRODUCTION

Pancreatic ductal adenocarcinoma (PDAC) is characterized by a low microvascular density (MVD) as compared to other tumor types [1]. Therefore, hypoxia inducible factor 1 alpha (HIF-1α) and vascular endothelial growth factor-A (VEGF-A) expression is increased and correlates with poor prognosis [2–3]. Another typical feature of PDAC is the presence of an intense fibro-inflammatory reaction, namely desmoplastic reaction (DR), responsible of an high intratumoral pressure and solid stress causing vasculature collapse [1, 4].

However, even though the anti-angiogenic treatments improved survival in subcutaneous and orthotopic pre-clinical models, the same treatments resulted ineffective in genetically engineered mouse models (GEMMs) of PDAC, as well as in clinical trials [5]. Differently from the transplantable models that have low stroma and pancreatic cancer cells (PCCs) are close to the vessels, GEMMs and human PDAC are characterized by a dense stroma, which is responsible of a high interstitial pressure and collapsed vessels with and impaired drug delivery.

This review will focus on the peculiar tumor angiogenesis and microenvironment in PDAC, and on the effects of these findings on the efficacy of anti-angiogenic clinical trials.

## ANGIOGENESIS AND TUMOR MICROENVIRONMENT IN PDAC

Different studies have demonstrated a relationship between microvascular density (MVD), tumor VEGF-A levels, and disease progression in PDAC [6–10]. The functional analysis of the tumor vasculature has demonstrated that vessels appear collapsed as a consequence of high interstitial pressure with a low delivery of small molecules [1, 4]. PDAC is characterized by a fibro-inflammatory reaction, namely DR, which consists in an abundant deposition of dense collagen types I and III bundles, hyaluronic acid and fibronectin, loss of basement membrane integrity, and invasion of malignant cells into the interstitial matrix associated with a disorganized vasculature characterized by vessels with variable diameters, abnormal multiple branching and disrupted interendothelial junctions [11–13].

DR is the result of a complex interplay between pancreatic stellate cells (PSCs) and PCCs. Co-colture of these two types of cells or the incubation of PCCs with PSCs supernatants results in a significant increase of release of endostatin by PCCs. Moreover, both PSCs and PCCs produce matrix metalloproteinase-12 (MMP-12) and cathepsin B to cleave endostatin from collagen XVIII. Endostatin increases hypoxia levels by inhibiting the angiogenesis and at the same time stimulates the secretion of MMPs by PSCs [14].

**Figure 1:**
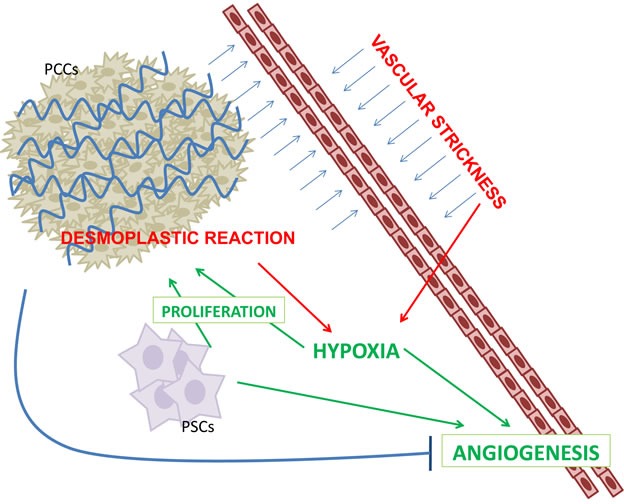
PDAC is characterized by a robust fibro-inflammatory response, namely desmoplastic reaction (DR) DR is the result of a complex interplay between pancreatic stellate cells (PSCs) and pancreatic cancer cells (PCCs). DR increased vascular strickness inducing hypoxia. Angiogenesis is both inhibited by DC and is enhanced by PSCs and hypoxia.

This hypoxic microenviroment, not only contributes to pro-fibrogenic activity of PSCs but stimulates PSCs to produce several angiogenic molecules, including VEGF, fibroblast growth factor-2 (FGF-2), platelet derived growth factor (PDGF), interleukin-8 (IL-8), MMP-9, and vasohibin-1, resulting in foci of angiogenesis in the peripheral areas of the tumor [14–15] (Figure 1). High levels of HIF-α increases, in turn, VEGF-A expression, and HIF-1α and VEGF-A not only contribute to PDAC aggressiveness by angiogenesis but also by a direct stimulation of tumor cell proliferation and metastatic capacity [2–3]. Stimulation of tumor cell proliferation and metastatic capacity through the regulation of the expression of actin-bundling proteins, MMPs and chemokine receptors, also occurs [16]. Furthermore, in PDAC other mitogenic and pro-angiogenic growth factors are over-expressed including transforming growth factor beta (TBF-β), hepatocyte growth factor, epidermal growth factor, and insulin like growth factor [17]. In particular, once activated, TGF-β receptors phosphorylate SMAD proteins to form complexes with transcription factor SMAD4, involved in the regulation of several genes which control angiogenesis and extracellular matrix remodeling [18].

## INFLAMMATION IN PDAC

Inflammatory cells contribute to the proliferative and invasive capacity of solid and hematological malignancies [19–21]. PDAC is characterized by an abundant inflammatory infiltration consisting in cancer-associated fibroblasts, macrophages, mast cells (MCs) and lymphocytes [12, 22]. MCs are now recognized as critical components of tumor stromal microenvironment. They are more numerous in PDAC than in benign pancreatic pathology and, at the same time, they are more numerous in PDAC than in the normal adjacent tissue [22–23]. In addition, PDACs with elevated numbers of infiltrating MCs and high MVD have a worse prognosis [24–25]. PCCs induce MCs and macrophages migration releasing chemoattractant molecules, such as colony-stimulating factor-1 and chemokine ligand 2 [26]. MCs express pro-angiogenic factors as VEGF-A, FGF-2, PDGF, tryptase, chymase, and MMPs promoting tumor growth [27–28]. Moreover, MCs promote PSCs proliferation releasing IL-13 and tryptase, and PSCs stimulate MCs proliferation [29].

Tumor-supportive M2-macrophages number is higher in PDAC than in benign pancreatic pathology and in normal tissue [22], and correlates with higher rate of metastasis and poor prognosis [30]. Inhibition of macrophage recruitment to the tumor microenvironment by targeting adhesion molecule integrin α4β1 or myeloid PI3Kγ resulted in a marked decrease of blood vessel formation in pancreatic cancer models [31].

## GENES INVOLVED IN PDAC ANGIOGENESIS

HIF-1α G1790A and C1772T single nucleotide polymorphisms appeared more frequently in PDAC, predicting higher risk for its development [32]. HIF-1α expression in PSCs results as a sensor of oxygen levels in pancreatic tissue, inducing an up-regulation of VEGF levels [15]. Moreover, PSCs improve angiogenesis by enhancing the levels of angiopoietin-1 and its receptor Tie-2 mRNA, both involved in the control of tumor angiogenesis, in PDAC microenvironment [15, 33].

Angiogenic gene signature has been assessed in PDAC analyzing the levels of 129 angiogenic genes from The Cancer Genome Atlas (TCGA). About 35% of PDACs show an up-regulation of pro-angiogenic genes, including FGFR-1, and VEGFR-1, −2 and −3, and of pro-inflammatory genes, such as IL-1B, IL-6, and JAK2, anti-inflammatory IL-10, as well as histone deacetylase-9, with a major involvement of TGF-β, which regulates the smad signal pathway [34]. Most of PDAC show loss of heterozygosity for SMAD4 locus, with 50% of PDAC having either homozygous deletion or mutational inactivation of the second allele. SMAD4 restoration inhibited both angiogenesis and extracellular matrix remodeling [18]. Conversely, SMAD4 re-expression in BxPC3 PCCs fails to suppress angiogenesis *in vivo* [35]. SMAD4 and nuclear SMAD4 expression correlates with high levels of CD31, the main endothelial cell-specific marker [34].

MicroRNAs (miRNA), play an important role in the regulation of gene expression in PDAC, including angiogenesis [36]. Hypoxia induces the expression of miRNA-21 in PDAC cells *via* HIF-1α allowing cells to avoid apoptosis [37], at the same time miRNA-21 acts as an inhibitor of endothelial cell proliferation and migration [38]. In a PDAC model, a lentiviral transported antimiRNA-21 improved tumor angiogenesis, improving the flow of gemcitabine with a synergistic antitumoral effect [39]. Whereas, MiR139 and miR200c resulted upregulated in primary endothelial cell cultures derived from PDAC patients, suggesting that they may improve tumor angiogenesis [40].

Although there has been some progress concerning angiogenesis gene signature in PDAC, the epigenetic regulation systems seem to be still poorly known, and might be used as a possible therapeutic target.

## PRE-CLINICAL ANTI-ANGIOGENIC APPROACHES IN PDAC

Several Authors targeted VEGF signaling using nude mouse models of human PDAC. Injection of PDAC antisense VEGF-A gene cells into athymic nude mice lead to an 80% decrease of tumor growth compared with mice injected with control cells [41]. Engineerization of Panc-1 cells and PK-8 cells to produce a soluble form of the decoy receptor of VEGF, inoculated in SCID mice, resulted in a PDAC model with both low MVD and tumor growth [42]. Ziv-aflibercept, a VEGF-Trap, decreased tumor MVD and suppressed cancer cell proliferation in an orthotopic model of PDAC [43]. More recently, curcumin analogues UBS109 and EF31 downregulated angiogenic factors such as HIF-1α, Hsp90, COX-2 and VEGF in xenograft models of PDAC showing antitumor and anti-angiogenic effects [44]. LY294002 (a PI3K inhibitor) in combination with gemcitabine and ionizing radiation, inhibited cancer cell growth, metastasis and angiogenesis targeting PI3K/MMPs/Ln-5γ2 signaling pathways in xenograft model in which vasculogenic mimicry occurs. [45]. Ginsenoside Rg3, a tetracyclic triterpenoid saponin, altered vasculogenic mimicry process in nude mouse xenografts of PDAC, downregulating the expression level of VE-cadherin, EphA2, MMP-2 and MMP-9 mRNA [46]. The simultaneous target of TGF-βR and JAK1 phosphorylase, using SB505124 and ruxolitinib suppressed endothelial activation in 3D co-cultures of PDAC and endothelial cells by suppressing proliferation and angiogenesis [34].

Experimental models are characterized by an high vascularity and the absence of desmoplastic reaction, as instead occurs in human PDAC. To avoid this pitfall, a GEMM of PDAC characterized by a dense stroma and collapsed vessels has been developed, using mutant Kras and p53 alleles in pancreatic cells (KPC) mice models [47]. In these models, sunitinib, targeting VEGF and PDGF receptors impair angiogenesis, but fail to reduce tumor burden [48]. Similarly, gemcitabine plus bevacizumab did not achieve a statistical increase of median (m) OS or progression free survival (PFS) [49].

As consequences of hypoperfusion, a reduced sensitivity to chemotherapy derives from a limitation of drug delivery due to reduced vascularization [1]. In this context, murine models have been developed in order to normalize the vasculature [50] or to reduce the pressure on the collapsed vessels, [1, 51]. The small molecule LB-100, an inhibitor of phosphatase 2A (PP2A), increased MVD in the PDAC xenograft model resulting in a higher chemotherapeutics delivery with improvement of objective response [50].

A recombinant human hyaluronidase conjugated with polyethylene glycol has been used in KPC mice PDAC models with a reduction of intratumoral hyaluronan and a significant improvement of tumor perfusion without an increase of MVD favouring chemotherapeutic delivery. KPC mice treated with gemcitabine together with this drug or gemcitabine alone, show a significantly increased response and mOS [1]. In a parallel similar study, Jacobetz et al demonstrate that hyaluronidase leads to microenvironment changes in endothelium with an increase of vascular permeability drug permeability, and an increase of survival in KPC mice treated with gemcitabine [4]. IPI-926, a derivate of cyclopamine, targeting Hedgehog pathway by inhibiting Smo, reduced the collagen-1 content and destroyed tumor-associated desmoplastic tissue, increased the MVD and concentration of gemcitabine, in KPC mice model [51]. Nonetheless, in contrast with this last study, in a Kre PDAC mice model, both the Shh (Sonic Hedgehog, a peculiar ligand of Hedgehog pathway) gene delection and Hedgehog targeting through IPI-926, reduced stromal content, but generated more aggressive PDAC with a high proliferation rate and an increased vascularity [52]. Furthermore, administration of DC101, an antibody blockingVEGFR-2, in Shh-deleted mice, reduces tumor proliferation inducing tumoral necrosis through angiogenesis inhibition. Moreover, inhibition of desmoplasia with depletion of myofibroblasts, resulted in transgenic mice with shorter survival and invasive and aggressive PDAC [53].

Despite the fact that many attempts to reduce the stroma to normalize tumor vascularization led to interesting results *in vivo*, most recent data have highlighted how this approach lead to a more aggressive phenotype with a lower survival. In parallel, although classic anti-angiogenic molecules, such as bevacizumab and sunitinib, has failed to induce tumor regression, the use of alternative angiogenetic targets, such as SB505124 and ruxolitinib of TGF-β type I receptor kinase and JAK1 phosphorylase, give more promising results in PDAC treatment.

## CLINICAL TRIALS OF ANTI-ANGIOGENIC THERAPY IN PDAC

Several phase II and III clinical trials have been conducted in PDAC using anti-angiogenic inhibitors. On the basis of a multicenter phase II trial in patients with metastatic PDAC which achieved a 21% ORR and a mOS of 8.8 months with the combination gemcitabine plus bevacizumab [54], Cancer and Leukemia Group B (CALGB) conduct a double-blind, placebo-controlled, randomized phase III trial of gemcitabine/bevacizumab *versus* gemcitabine/placebo in advanced PDAC. Five hundred and thirty-five patients were enrolled to receive gemcitabine at 1,000 mg/m^2^ over 30 minutes on days 1, 8, and 15 every 28 days and bevacizumab at 10 mg/kg or placebo on days 1 and 15 every 28 days. In spite the promising results of the phase II trial, the addition of bevacizumab to gemcitabine did not improve mOS of 5.8 and 5.9 months, with a not statistically improved mPFS of 3.8 and 2.9 months for combination arm and gemcitabine alone arm, respectively. The only statistically significant differences in grades 3 and 4 toxicity regarded hypertension (10% v 3%; *P* < .001) and proteinuria (5% v 1%; *P* = .002) [5]. The Authors imputed the different results between the two phase trials to the different selection of patients, i.e. a better PS in the phase II study.

A subsequent study, with the aim to identify predictive biomarkers of response to bevacizumab-containing regimen in PDAC has been conducted using serum from patients enrolled in the CALGB 80303 trial. One hundred and fifty-six proteins were quantified and authors selected histidine-rich glycoprotein (HRG) and complement factor H (CFH) as possible predictive markers. Unfortunately, there was no evidence for interaction with bevacizumab and HRG, but there was some evidence for a weak positive correlation of HRG with OS (τ = 0.11 [0.03, 0.19]; *P* < .01). CFH was found to be neither a predictive nor a prognostic factor for OS [55]. Subsequently, on the same setting of patients, three markers predictive for bevacizumab response were identified: VEGF-D, SDF1, and Ang-2. In particular, low levels of VEGF-D were predictive to benefit from bevacizumab plus gemcitabine arm, whereas, below median levels of both Ang-2 and SDF1 predicted for greater benefit in the placebo group [56]. However, the same authors assert the need to evaluate these markers in a larger sample in order to select the highest number of positive and negative predictive markers of response to anti-angiogenic treatment.

From the evidence that simultaneous inhibition of EGFR and VEGFR leads to better target angiogenesis, a phase III trial tested the use of bevacizumab added to the association gemcitabine-erlotinib. Three hundred and one PDAC patients were randomly assigned to receive gemcitabine (1,000 mg/m(2)/week), erlotinib (100 mg/day), with or without bevacizumab (5 mg/kg every 2 weeks). Despite a good safety profile and a better significant PFS (HR 0.73;*P* = .0002) of the triplet schedule, the addition of bevacizumab did not show a statistically significant improvement in terms of OS (7.1 and 6.0 months in the bevacizumab and placebo arms, respectively, HR 0.89; *P* = .2087) [57]. Similarly negative results were achieved in phase III trials using combination of gemcitabine with anti-angiogenic agents such as axitinib [58], sorafenib [59], and ZIV-aflibercept [60]. In a phase II randomized trial, sunitinib, a tyrosine kinases inhibitor, compared to observation alone showed a PFS at six months of 22.2% and 3.6%, a 2 years OS of 22.9% and 7.1%, respectively, in the maintenance therapy after a gemcitabine -based first line [61]. Even so, a not significant superiority was achieved for the combination of Sunitinib and Gemcitabine in a randomized phase II trial in first line locally advanced or metastatic PDAC [62].

Moreover, Elpamotide, a peptide VEGFR-2 vaccine inducing a cellular immune response against VEGFR-2 expressing endothelial cells, did not improved mOS or PFS compared to gemcitabine alone, although the subgroup that showed severe side effects at the injection site apparently had a better outcome [63]. Probably the failure of anti-angiogenesis could depend on the absence of predictors of response, moreover, only 35% of PDAC seems to have an angiogenic phenotype [34, 64]. Ramucirumab, a recombinant fully human monoclonal antibody directed against human VEGFR-2, is under investigation in a phase II study, evaluating the efficacy and safety of FOLFIRINOX plus ramucirumab (Arm A) *vs*. FOLFIRINOX plus placebo (Arm B) in 94 subjects with advanced.

As mentioned above in the pre-clinical studies, lack of response may derive from high interstitial pressures and collapse of tumor vasculature. In fact, targeting stromal microenvironment elements could be an efficient therapeutic strategy in addition to classical and new chemoterapic agents [1]. The possible therapeutic role of PEGPH20, has been recently investigated in a randomized phase II trial. The study enrolled untreated patients with metastatic PDAC to receive nab-paclitaxel and gemcitabine (nab-paclitaxel 125 mg/m^2^ plus gemcitabine 1000 mg/m^2^ given IV x1/week 3/4 weeks per cycle) combined with PEGPH20 (3ug/kg IV x 2/week for cycle 1 and weekly for cycle 2 and beyond, PAG) or placebo (AG). Following an initial clinical withdrawal for the evidence of several thromboembolic events (29% and 15% for PAG and AG, respectively) the trial was started again with a prevention treatment with low molecular weight heparin, and concluded that in hyaluronan-high expression patients receiving PAG and AG, the ORR was 52% *vs* 24% respectively (*P* = .038), while there was no difference in 37% *vs* 38% hyaluronan-low expression patients. Moreover PFS was increased in patients with hyaluronan-high expression, 9.2 and 4.3 for PAG and AG, respectively, and a there was a positive trend in OS [66]. On the basis of these results, a phase III trial of PAG has been started [67]. Based on encouraging preclinical data in Hedgehog signal role inhibition in tumor-associated stroma [51], IPI-926, an oral Hedgehog inhibitor, was evaluated in combination with FOLFIRINOX (5-fluorouracil, leucovorin, irinotecan, oxaliplatin) in a multicenter phase Ib study. Patients were treated with once-daily IPI-926 plus FOLFIRINOX at 3 + 3 dose escalation design. The combination was generally well tolerated, with common treatment-related adverse events such as liver function test abnormalities, neuropathy, nausea/vomiting, and diarrhea. Patients presented a promising ORR of 67%, with evident decline of CA19-9 levels [68]. Unfortunately, a phase II trial of IPI-926 plus gemcitabine was closed early due to an initial detrimental effect of this combination [69]. Furthermore, the MMP inhibitor marimastat was tested in patients with PDAC, based on the data that aberrant MMP expression is observed in this neoplasm. Bramhall et al. designed a phase III randomised study on 239 PDAC patients to compare orally administered marimastat in combination with gemcitabine to gemcitabine alone. There was no significant difference in OS between combination and gemcitabine plus placebo arm (*P* = 0.95), with a 1-year survival of 18% and 17%, respectively. Also no significant advantage was seen in ORR (11 and 16% respectively), in PFS (*P* = 0.68) and in time to treatment failure (*P* = 0.70) between the treatment arms [70].

In the future, the PDAC patients will be selected for the use of anti-angiogenic therapy through the angiogenetic signature. Moreover, the target of stroma through Hedgehog inhibitors or hyaluronidase enzymes together with actual standard therapies will plays a key role in the treatment of this malignancy. The main clinical trials are in progress are summarized in Table 1.

**Table 1:** Main clinical trials ongoing in PDAC targeting angiogenesis/stroma

Targeting PDAC angiogenesis					
Setting	Phase	Design	Mechanism of action	Primary endpoints	Trial identification number
I line	I	Ruxolitinib plus gemcitabine plus nab-paclitaxel	Janus-associated kinase 1 (JAK1) and JAK2 inhibition	Safety	NCT01822756
I line	I	Gemcitabine plus nab-paclitaxel plus GS-5745	MMP9 inhibition	Safety	NCT01803282
I line	I/II	5FU plus nab-paclitaxel plus bevacizumab plus calcium leucovorin plus oxaliplatin	Inhibition of vascular endothelial growth factor A	Dose limiting toxicities (Phase 1); 1 year survival rate (Phase II)	NCT02620800
Resectable	II	Gemcitabine plus bevacizumab plus external beam radiotherapy 3 Gy/fraction utilizing a 95% isodose field over 10 consecutive weekdays	Inhibition of vascular endothelial growth factor A	R0 resection rate/rate of complete pathologic response after resection	NCT00557492
II line	II	Regorafenib plus gemcitabine	Dual targeted VEGFR2-TIE2 tyrosine kinase inhibition	PFS	NCT02383433
II line	II	Regorafenib	Dual targeted VEGFR2-TIE2 tyrosine kinase inhibition	PFS	NCT02080260
II line	II	Regorafenib	Dual targeted VEGFR2-TIE2 tyrosine kinase inhibition	2-months progression free survival rate	NCT02307500
I line	II R	Gemcitabine plus TL118 vs gemcitabine	TL118 is a drug formed by four molecules: cyclophosphamide, diclofenac, sulfasalazine, and cimetidine, angiogetic inhibitors through anti-inflammatory mechanisms	Disease control rate	NCT01509911
I line	II R	mFOLFIRINOX plus ramucirumab	VEGF Receptor 2 inhibition	PFS	NCT02581215
II line (after gembitabine)	IIR	Capecitabine plus ruxolitinib vs capecitabine	Janus-associated kinase 1 (JAK1) and JAK2 inhibition	OS	NCT01423604
II line	III	Capecitabine plus ruxolitinib vs capecitabine	Janus-associated kinase 1 (JAK1) and JAK2 inhibition	OS	NCT02119663
**Targeting PDAC stroma**					
**Setting**	**Phase**	**Design**	**Mechanism of action**	**Primary endpoints**	**Trial identification number**
I line	I	mFOLFIRINOX plus IPI-926	Hedgehog pathway inhibitor	Maximum tolerated dose (MTD)	NCT01383538
I line	I	PEGPH20 plus cetuximab	Destruction of the stroma through the cleavage of hyaluronan	Safety	NCT02241187
I line	I II R	PEGPH20 mFOLFIRINOX plus PEGPH20 vs mFOLFIRINOX	Destruction of the stroma through the cleavage of hyaluronan	MTD/safety OS	NCT01959139
I line	II R	PEGPH20 plus nabpaclitaxel plus gemcitabine vs nabpaclitaxel plus gemcitabine	Destruction of the stroma through the cleavage of hyaluronan	PFS; Evaluation of the thromboembolic events	NCT01839487
Borderline resectable	II R	Gemcitabine plus nab-paclitaxel plus PEGPH20 vs gemcitabine plus nab-paclitaxel	Destruction of the stroma through the cleavage of hyaluronan	Pathologic complete response; Clinically relevant pancreatic fistula	NCT02487277
I line	III	Gemcitabine plus nab-paclitaxel plus PEGPH20 vs gemcitabine plus nab-paclitaxel	Destruction of the stroma through the cleavage of hyaluronan	PFS OS	NCT02715804

## CONCLUDING REMARKS

The hypothesis that the peculiar stroma is responsible of chemoresistance in PDAC explains the low efficacy of anti-angiogenic agents in PDAC treatment. On the other hand, this biological property of PDAC microenvironment has led to suggest the depletion of tumor stroma as a strategy for PDAC treatment. However, this approach seems contradictory because in some GEMM studies stromal depletion with increased tumor vascularity and drug diffusion resulted efficacious, resulting in increasing survival. In the meantime, other studies have demonstrated that increased vasculature correlates with disease progression. In this context, strategies aimed to achieve a more precise and efficacious modulation of desmoplasia and tumor vascularity in PDAC are necessary.
